# Berries to Go: Distinct Passerine Spring Migration Frugivory at a Main Mediterranean Stopover Site

**DOI:** 10.1002/ece3.72239

**Published:** 2025-10-02

**Authors:** Julia Slezacek, Benjamin Kostner, Chiara Agabiti, Massimiliano Cardinale, Leonida Fusani

**Affiliations:** ^1^ Department of Interdisciplinary Life Sciences, Konrad Lorenz Institute of Ethology University of Veterinary Medicine Vienna Vienna Austria; ^2^ Eurac Research Institute for Regional Development Bozen Italy; ^3^ Department of Biology University of Pisa Pisa Italy; ^4^ Department of Aquatic Resources, Marine Research Institute Swedish University of Agricultural Sciences Lysekil Sweden; ^5^ Department of Behavioral and Cognitive Biology University of Vienna Vienna Austria

**Keywords:** facultative frugivory, passerine spring migration, *Prasium majus*, refuelling, *Rhamnus alaternus*, stopover

## Abstract

Many animals show phenotypic flexibility in response to a seasonal environment. Especially migratory birds have been found to exhibit striking physiological and behavioural adaptations to overcome the negative impacts of environmental seasonality. Migratory songbirds often show extreme changes in feeding physiology and behaviour before embarking on a migratory flight, including predominantly insectivorous species switching their diet preference to a frugivorous one before autumn migration. Yet, little is known about frugivory during spring migration in temperate zones. In this paper, we report that five songbird species forage on the fruits of two Mediterranean plants, *Prasium majus* and *Rhamnus alaternus,* during spring stopover in the Tyrrhenian Sea. Analyses of faecal content showed that fruits of *P. majus* were generally preferred, with garden warblers ( *Sylvia borin* ) having the highest percentage of faecal samples containing seeds of both plants. Availability of ripe *P. majus* fruits increased over the sampling season and correlated positively with the number of faecal samples containing seeds. Our findings reveal a relevance of fruit at a temperate zone stopover site during spring migration for five passerine species. Frugivory during spring migration may represent an easy means for birds to acquire macronutrients, micronutrients and water. This may be especially important at resource‐poor stopover sites and may aid birds' continuation of the northward flight towards their breeding grounds in a timely manner.

## Graphical abstract

We report that five songbird species forage on the fruits of two Mediterranean plants, *Prasium majus* and *Rhamnus alaternus* , during a main spring stopover in the Tyrrhenian Sea. Analyses of faecal content showed that fruits of *P. majus* were generally preferred and an increase in *P. majus* ripe fruit availability over the sampling season was positively correlated with the number of passerine faecal samples containing seeds.

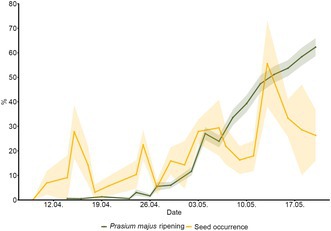

## 1 Introduction

In temperate zones, life is shaped by the seasonality of the environment, with adaptations to characteristic weather and temperature changes during the annual cycle. In response to seasonal patterns, animals as well as plants have evolved periodic phenotypic flexibility, with physiological, anatomical, morphological and behavioural adaptations to predictable environmental changes (Bradshaw 1965; reviewed in: West‐Eberhard 1989; Gratani 2014; Fusco and Minelli 2010). In animals, migration is a common strategy to tackle seasonal food depletions, avoid unfavourable weather conditions, and/or increase reproductive success (reviewed in Alerstam et al. 2003). Several striking adaptations to a migratory lifestyle can be observed in birds, which typically undergo drastic changes concerning their feeding strategy and digestive physiology before and during migration (Jehl Jr 1997; Piersma and Gill Jr 1998; reviewed in McWilliams and Karasov 2005). In preparation for a migratory flight, birds need to build up large energy deposits. Lipids account for about 95% of the required migratory fuel and proteins catabolised from muscle tissue and digestive organs for 5% (reviewed in Jenni and Jenni‐Eiermann 1998). The accumulation of fuel deposits before migration may result in an increase of up to 100% of the individual body weight for some species and is facilitated by hyperphagia (i.e., overeating) (Odum 1960; Maillet and Weber 2006), which is accompanied by an enlargement of digestive organ size required to process the increased influx of food (reviewed in McWilliams and Karasov 2005).

Especially in autumn, many bird species have been found to alter their diet preference from predominantly insectivorous to largely or even completely frugivorous during migratory hyperphagia (Berthold 1976; Herrera 1984; Parrish 1997; also reviewed in Bairlein and Simons 1995). Fruits consumed by birds are usually rich in carbohydrates and fibres (Smith et al. 2007, 2015) and often contain a high proportion of water (Ferns 1975; Berthold 1976; Snow and Snow 2010). Besides their macromolecular content, fruits offer additional beneficial properties by typically being rich in secondary plant compounds (i.e., micronutrients), such as vitamins, phenolic acids, flavonoids or tannins, known for their antioxidant and/or antimicrobial capacities (reviewed in Haminiuk et al. 2012; Skrovankova et al. 2015; Puupponen‐Pimiä et al. 2001). Fruit consumption during migration is thought to represent an evolutionary adaptation to compensate for the autumnal decline in insect abundance: Fruits are superabundant in autumn and are an ‘easy prey’ because birds do not have to spend time and energy to hunt for them thanks to their usually patchy distribution (Berthold 1976). Yet, fruits have a poorer nutritional value with lower fat content and especially low protein content compared to insect prey—a reason for seasonal frugivory sometimes having been termed the ‘frugivory paradox’ (Bairlein 2002). An answer to resolve this paradox has, however, been offered by Langlois and McWilliams (2010) who demonstrated that the hyperphagic state associated with migration may reduce birds' physiological demand for dietary protein, thereby allowing them to rely more heavily on fruit despite their relatively low protein content. In fact, migratory birds are able to maintain their body condition on a fruit diet: garden warblers ( *Sylvia borin* ), for example, can maintain or even increase their body fat on a pure fruit diet after an acclimation of only a few days, despite an initial decrease in body mass (Simons and Bairlein 1990). Additionally, field studies conducted during autumn migration found that birds caught at stopover sites with available fruit had a higher body mass and greater mass gain during refuelling than birds caught at sites without fruits (Thomas 1979; Smith and McWilliams 2010; Smith et al. 2015). Finally, captive garden warblers provided with a mixed fruit and insect diet had an increased body mass gain compared to conspecifics kept on an exclusive insect diet (Bairlein 2002).

While frugivory during autumn migration is well documented across many bird species and regions, much less is known about its role during spring migration—the second, equally critical half of the migratory journey—when fruits are generally scarce outside tropical and subtropical regions. Whether and to what extent migratory songbirds rely on fruit during spring migration remains thus largely unexplored. Gaining insight into this question contributes to a better understanding of migration ecology and the flexibility of avian foraging strategies across seasons. Mediterranean islands are important stopover points for passerines crossing the Mediterranean Sea during spring migration (Spina et al. 1993). One of the main Mediterranean spring stopover sites, the island Ponza, located in the Tyrrhenian Sea and along one of the main migration routes for European passerines (Maggini et al. 2021), is thought to be a nutrient‐poor island that is used by passerines mostly for resting (Maggini, Trez, et al. 2020). In this study, we explored the potential contribution of fruits to the diet of six different passerine species that stop over on Ponza during spring migration. Specifically, we investigated: (1) which plant seeds occur in the faecal matter of six passerine species; (2) at which rate seeds occur in faecal matter and whether there are differences in faecal seed occurrence between species. We also studied the ripening of fruits of the different plants producing the majority of seeds found in the faecal matter of each of the focal passerine species. Our findings offer insights into the consumption, diversity, and seasonal availability of wild fruits at a key Mediterranean stopover site and their potential role as a dietary component during avian spring migration.

## 2 Material and Methods

We collected all data on the island of Ponza in May 2019 and March–May 2021. Ponza Island is the biggest island of the Pontine archipelago in the Tyrrhenian Sea and is located about 50 km off the western coast of mainland Italy (40°55′ N, 12°58′ E), along one of the main avian migratory routes for western Palearctic migrants. The island's location and the existence of a local ringing station, operating each spring, have made Ponza a strategic point for studies of avian migration (reviewed in Lupi et al. 2019; Ferretti et al. 2021).

### 2.1 Determination of Seed Presence in Faecal Samples

We carried out a preliminary collection of faecal seed samples from wild garden warblers ( *Sylvia borin* ) during the field season of 2019 (07.05.2019–17.05.2019). During ringing activities, which spanned from 06:00 to 21:00, we used clean and marked cotton bird bags to transport garden warblers that had been trapped in mist nets to the ringing station. Within 30 min from capture, birds were ringed and measured using standard procedures (Bairlein 1995). Body mass was measured with an electronic scale with a precision of ±0.1 g. Abdominal fat and pectoral muscle size were scored on a 0–8 scale (fat) or a 0–3 scale (muscle) (Bairlein 1995), and scoring was performed by the same ringer in both seasons to exclude a possible interobserver bias. All birds were released immediately after ringing procedures.

After releasing the birds, we checked each bird bag and emptied the faecal contents onto a sheet of clean paper. We collected all seeds and intact fruits found in the faecal matter and categorised seeds and berries based on their morphology. Furthermore, we counted the number of seeds and fruits in each bag. We collected all seeds and berries retrieved from a bag in Eppendorf tubes, labelled with the individual ring number of a bird and stored all samples at −20°C.

During the spring ringing season of 2021, we again collected faecal seed samples following the protocol of 2019 but extended collection to six passerine species: garden warbler, Eurasian blackcap ( *Sylvia atricapilla* ), common whitethroat (*Curruca communis*), icterine warbler ( *Hippolais icterina* ), common redstart ( *Phoenicurus phoenicurus* ), and whinchat ( *Saxicola rubetra* ). Furthermore, the sampling period was longer than in 2019 and lasted from 09.04.2021 to 20.05.2021.

### 2.2 Identification of Fruiting Plants on Ponza Island

**FIGURE 1:**
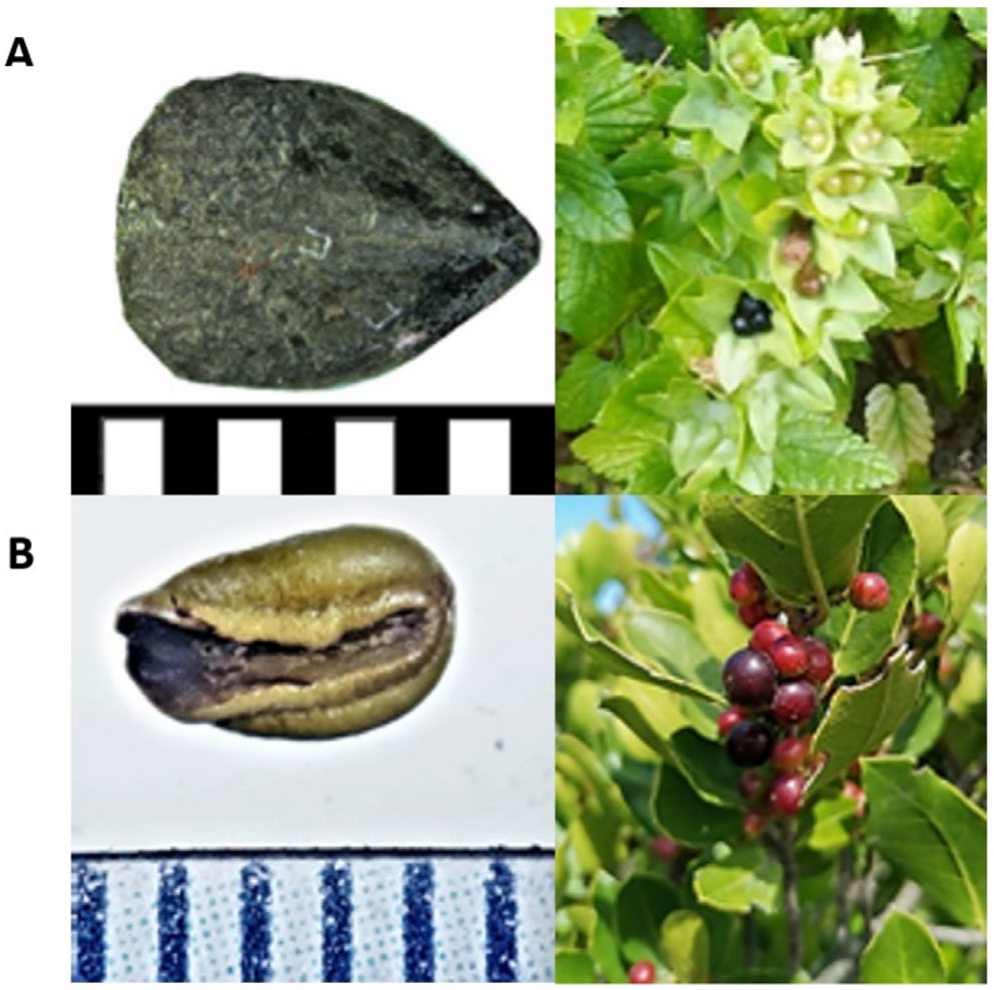
*Prasium majus* (A): Seeds are black, triangular‐like shaped, pointed on one side and rounded on the opposite one (panel A left; scale = mm). Unripe fruits are green, and they darken to black when ripe (panel A right). *Rhamnus alaternus* (B): Seeds are light green to brown, symmetrical‐ovoidal shaped with a deep longitudinal groove on one side that develops in the drying process. When moist, the surface is smooth (panel B left; scale = mm). Unripe fruits are red to brown and darken to black when ripe (panel B right).

To identify sources of fruits on Ponza in springtime, we performed an exploratory search of candidate fruiting plants in six different locations across the island: area around Punta Corta and Belvedere della Madonnina (40°55′21.4″ N 12°57′50.6″ E), area around Piscine Naturali (40°55′08.8″ N 12°57′43.1″ E), area around the CISCA (Centro Italiano Studi per la Conservazione e l'Ambiente; Italian Study Centre for Environmental Conservation) ringing station in Le Forna (Stazione di Inanellamento di Ponza; 40°55′11.7″ N 12°58′00.7″ E), area around Forte Papa (40°55′44.2″ N 12°57′58.1″ E; 40°55′44.3″ N 12°58′11.8″ E), area around Punta d'incenso (40°55′57.0″ N 12°59′31.8″ E), and area around Il Semaforo (40°53′06.9″ N 12°57′23.6″ E). We selected plants which carried fruits with seeds resembling those we previously found in bird faeces and identified two plants producing most seeds present in faecal matter: *Prasium majus* and *Rhamnus alaternus* (Figure 1; Table 1).

**TABLE 1:** Overview of the sampled bird species and the percentage of occurrence of seeds of *P. majus* exclusively, *R. alaternus* exclusively, seeds of both plants and the total percentage of faeces containing seeds ( *P. majus* and/or *R. alaternus* ) in general.

	Bird species	% Birds consuming only *P. majus*	% Birds consuming only *R. alaternus*	% Birds consuming both	% Total birds consuming any fruit
*Muscicapidae*	*Phoenicurus phoenicurus* (Common redstart)	0.00	0.00	0.00	0.00
	*Saxicola rubetra* (Whinchat)	5.06	1.27	0.00	6.33
*Sylviidae*	*Sylvia atricapilla* (Eurasian blackcap)	22.22	0.00	0.00	22.22
	*Curruca communis* (Common whitethroat)	12.55	0.00	0.37	12.92
	*Sylvia borin* (Garden warbler)	50.87 (2019) 20.00 (2021)	15.61 (2019) 15.56 (2021)	10.98 (2019) 6.67 (2021)	77.46 (2019) 42.22 (2021)
*Acrocephalidae*	*Hippolais icterina* (Icterine warbler)	5.26	5.26	0.00	10.52

*Note:* Garden warbler data include samples collected in the seasons 2019 and 2021. Samples for all other species were only collected in the season of 2021.

*Prasium majus* belongs to the *Lamiaceae* family and is known as Mediterranean prasium or white hedge‐nettle. On Ponza, fully ripe fruits are typically present from the end of April to the beginning of May. *Rhamnus alaternus* is an evergreen shrub of the *Rhamnaceae* family, also known as Mediterranean buckthorn. Its fruits are usually only present from June to the beginning of July (Aronne and Wilcock 1994), however, we detected few ripe fruits already in May on Ponza. Both plants are endemic species of the Mediterranean scrub ecosystem. Their seeds have a characteristic shape and colour that make their field identification possible (Figure 1).

To confirm seed identity in bird faeces, we compared the seeds collected from both fruiting plants and faecal samples using stereomicroscopes at the Konrad Lorenz Institute of Ethology (KLIVV) in Vienna and the European Academy of Bolzano (EURAC).

### 2.3 Phenological State of Fruiting Plants

**FIGURE 2:**
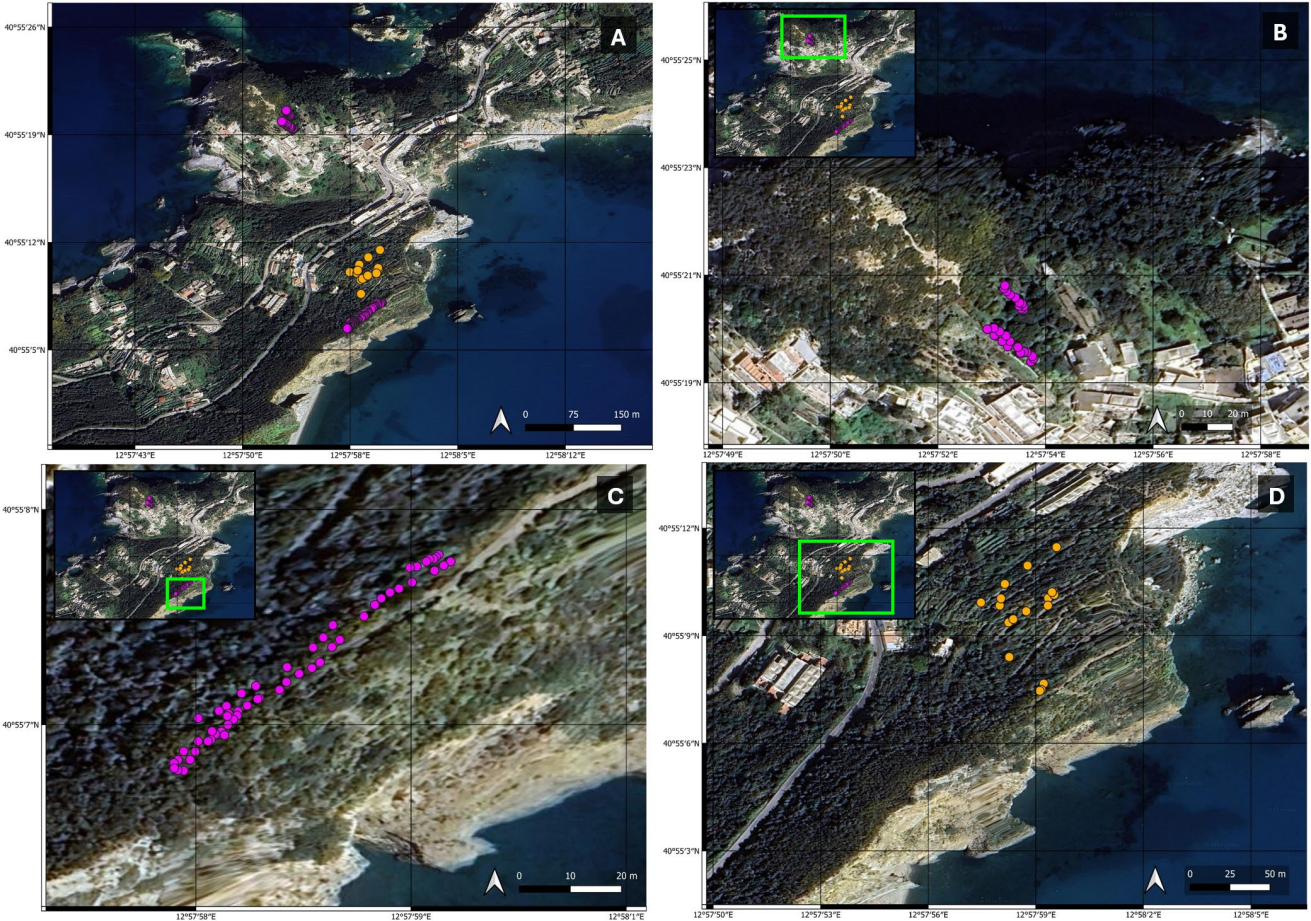
Plants selected for data collection in 2021: Overview of transects (panel A); *R. alaternus* plants (orange points; panel A and D) and *P. majus* plants (purple points; Madonnina transects, panel B.; Cima transects, panel C).

In 2021, we collected data on the fruiting phenology of *P. majus* and *R. alaternus* , with the main aim of estimating the ripening stage of fruits during passerine stopover. We created four transects in two different locations for *P. majus* plants: 40°55′22″ N 12°57′51″ E (‘Madonnina transect’; Figure 2B) and 40°55′7″ N 12°57′59″ E (‘Cima transect’ at the ringing station; Figure 2C). Sampling locations were chosen in proximity to or at the ringing station, to represent ripe fruit availability at the same location in which the birds were caught. The transects were 3 m wide and 15–60 m long, with transect length, spacing, and orientation varying between sampling locations due to the difficult nature of the terrain in most natural places on the island. In locations with dense shrubs and rocky, steep terrain, the transects were shorter (Figure 2B) whereas the paths created for mist nets at the ringing station allowed for a longer transect (Figure 2C). In the selected transects, all *P. majus* plants along the width and length of the transect were registered and numbered. For *R. alaternus* plants, we did not create transects due to the patchy appearance of the plant but rather numbered all plants located around the mist nets at the ringing station (Figure 2D). In the *P. majus* transects, we selected a total of 82 plants and for *R. alaternus* we selected 17 plants to be checked at regular intervals for fruit ripening stage. We labelled two branches on each of the selected bushes to monitor fruit ripening throughout the season. For this, we marked every plant and every selected branch with a plastic label with a unique ID. We checked plants every 2–3 days during the months of April and May (14.04.2021 to 20.05.2021), thereby collecting variables concerning the entire plant (occurrence of fruits) and branches (number of ripe and unripe fruits; only fully ripe, black fruits, were considered as ‘ripe’ and all other fruits as ‘unripe’). The same observer estimated a total mean ripening stage of fruits on each plant by taking into account the counted ripe and unripe fruits and the height of plants to approximate the ratio of the total plant volume which the selected branches represented. The protocol for fruit monitoring was modified after Herrera (1984).

### 2.4 Statistical Analyses

We performed statistical analyses in R Studio version 2024.4.1.748 (Posit Team 2024) and R version 4.2.1 (R Core Team 2021). For statistical analyses, we divided the data concerning bird faecal samples into two subsets: one subset consisted of data on garden warblers of both sampling years but limited to the period from May 7 until May 16 to allow comparison between the 2 years. The second dataset consisted of all data on all species collected in 2021.

To obtain birds' body conditions, we performed principal component analyses (PCA) for each species: PCAs included individual subcutaneous fat score, pectoral muscle size, and the residuals of a linear model between body mass and third primary covert length to correct body mass by individual body size (Maggini et al. 2013). We extracted principal component 1 with eigenvalues > 1 and included it as an index of body condition in our data set (= individual body condition). Detailed PCA outputs, including PCA eigenvalues, loadings, and scree plots for each species can be found in Section 1 of the Appendix S1 (Figures A1–A6).

We performed generalised linear models (glm; native *stats* package) with a binomial error structure (*P. majus/R. alaternus* seed presence vs. seed absence) for all analyses regarding *P. majus* or *R. alaternus* seed occurrence in bird faeces. For garden warbler data, the models either contained *P. majus* or *R. alaternus* seed occurrence as the dependent variable and sampling year, day in May, and individual body condition as independent variables. Body condition dependency of avian movements across the migratory season is a common phenomenon—for example, individuals with larger fuel depots often migrate earlier than individuals in poorer body condition (Schmaljohann and Naef‐Daenzer 2011; Smolinsky et al. 2013; Smith and McWilliams 2014). We therefore made sure that the variables body condition and day of May were not correlated by calculating a *Spearman* correlation coefficient and visually inspecting the data before including these variables in our models. To compare *P. majus* seed occurrence between species, we again used *P. majus* seed occurrence as the dependent variable and species (excluding common redstart as there was no sample containing *P. majus* seeds), sampling date (formatted as Julian date), and individual body condition as independent variables. We again made sure that there is no correlation between body condition and sampling date by means of a *Spearman* correlation coefficient and by plotting and visually inspecting the data before running the model. We performed a post hoc test with Tukey correction (*emmeans* package; Lenth 2024) to show differences in *P. majus* seed occurrence between the tested species. We did not model *R. alaternus* seed occurrence between species, as the sample size of faeces containing *R. alaternus* seeds in the different species was too small. We tested correlations between sampling date and the estimated percentage of ripe *P. majus* fruits and the percentage of their seed occurrence in faecal matter by performing correlation tests using the *Spearman* method. Because faecal samples and fruit ripening were sampled on alternating days, we used the *data.table* package (Barrett et al. 2025) to match nearest sampling dates of seed occurrence and ripening stage to allow performing a *Spearman* correlation test between the variables.

We checked glm model fit using diagnostic plots provided by the *DHARMa* package (Hartig 2024). The diagnostic plots confirmed no significant deviations from model assumptions and can be found in Section 2 of the Appendix S1 (Figures A7–A14). Additionally, variance inflation (VIF) was checked for all variables in models (VIFs ≤ 1.5 for all models).

## 3 Results

We recorded seed occurrence of *P. majus* and/or *R. alaternus* in faecal samples of all focal species, except in the common redstart, which did not contain seeds of either plant in its faeces (Table 1). Occasionally, we also found intact ripe *P. majus* fruits and unripe fruits of *R. alaternus* in bird bags, probably regurgitated. The highest number of *P. majus* seeds in one faecal sample amounted to 31 seeds, and the highest number of *R. alaternus* seeds in a single sample amounted to 11 seeds. Both samples stemmed from garden warblers.

**FIGURE 3:**
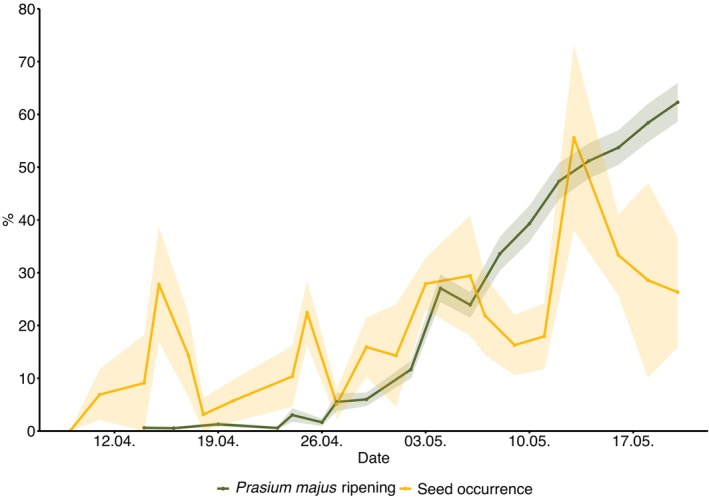
Percentage of *P. majus* seed occurrence in individual faecal samples of five different passerine species during spring migration stopover in the field season of 2021 on Ponza island, depicted by the yellow line (*n* = 676 individual faecal samples). The green line depicts the estimated percentage of ripe *P. majus* fruits throughout the sampling season (*n* = 1312 plant recordings). Ribbons around lines represent standard errors.

We did not find significant differences in the frequency of *P. majus* seed presence between samples from garden warblers, blackcaps, and whitethroats (Table 2). Whinchats and icterine warblers had lower faecal *P. majus* seed occurrence compared to samples from garden warblers and there was a trend of whinchats and icterine warblers having lower faecal *P. majus* seed occurrence compared to blackcaps (Table 2). In addition, sampling date had a positive influence on *P. majus* seed occurrence in bird faeces (*β* = 0.04, SE = 0.01, *p* < 0.01), signifying that more samples containing seeds could be found later in the season (Figure 3). Birds' body condition was negatively associated with the occurrence of *P. majus* seeds in bird faeces (*β* = −0.21, SE = 0.08, *p* < 0.01). There was no significant relationship between birds' body condition and sampling date (*r* = 0.16).

**TABLE 2:** *P. majus* seed occurrence between five common passerine species caught during 2021 spring migration stopover on Ponza island (*n* blackcap = 18, *n* garden warbler = 270, *n* icterine warbler = 38, *n* whinchat = 79, *n* whitethroat = 271).

Bird species	*β*	SE	*z*	*p*
Garden warbler—Blackcap	−0.34	0.63	−0.54	0.98
Garden warbler—Whitethroat	0.52	0.27	1.95	0.29
Garden warbler—Whinchat	1.67	0.54	3.08	0.02*
Garden warbler—Icterine warbler	2.33	0.76	3.05	0.02*
Blackcap—Whitethroat	0.86	0.61	1.42	0.62
Blackcap—Whinchat	2.01	0.78	2.57	**0.08**
Blackcap—Icterine warbler	2.67	0.99	2.69	**0.05**
Whitethroat—Whinchat	1.15	0.55	2.09	0.23
Whitethroat—Icterine warbler	1.81	0.80	2.27	0.16
Whinchat—Icterine warbler	0.66	0.92	0.72	0.95

*Note:* * and bold numbers in the column ‘*p*’ mark a significant difference, whereas bold numbers only mark a marginally significant difference.

Abbreviations: *β*, *β*‐coefficient; *p*, *p* value; SE, standard error; *z*, *z*‐value.

**FIGURE 4:**
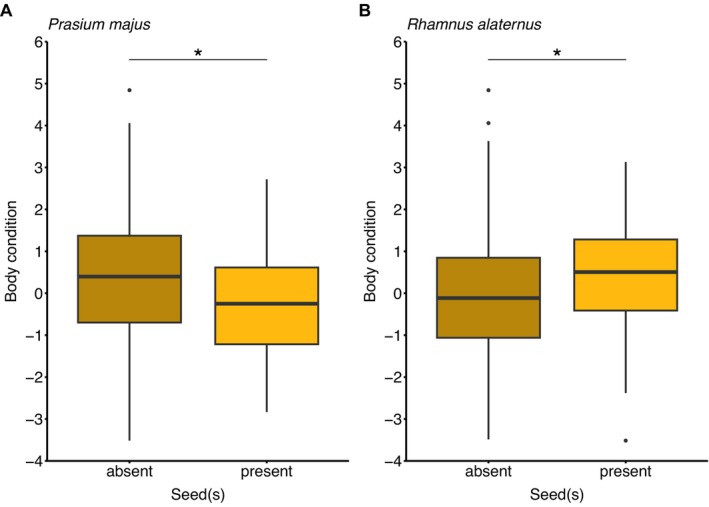
*P. majus* (Panel A) and *R. alaternus* (Panel B) seed occurrence in garden warbler faeces in relation to individual body condition. Samples were collected between May 7th and May 16th in the years 2019 and 2021 (*n* = 289).

In the model exclusively looking at garden warblers and *P. majus* seed occurrence, individual body condition was negatively related to seed occurrence in faecal matter implying that we found seeds of *P. majus* less often in faeces of birds with higher body condition (*β* = −0.28, SE = 0.09, *p* < 0.01; Figure 4A). When modelling *R. alaternus* seed occurrence in garden warblers, we found the opposite relationship between individual body condition and seed occurrence, meaning that birds with a higher body condition were more likely to feed on *R. alaternus* (*β* = 0.26, SE = 0.10, *p* < 0.01, Figure 4B). Median body condition of garden warblers was −0.08 in the season of 2019 and 0.36 in 2021. There was a significantly lower occurrence of *P. majus* seeds in faeces of garden warblers in 2021 compared to 2019 (*β* = −0.71, SE = 0.13, *p* < 0.001; Table 1). In contrast to *P. majus* seed occurrence, there was no significant difference in *R. alaternus* seed occurrence between years (*β* = −0.17, SE = 0.14, *p* = 0.22). Garden warbler faeces contained significantly more *P. majus* seeds when the month of May progressed (*β* = 0.13, SE = 0.04, *p* < 0.01), yet no such relationship was evident for the occurrence of *R. alaternus* seeds (*β* = 0.00, SE = 0.04, *p* = 0.89). Garden warbler body condition was not correlated with sampling date (*r*_overall_ = 0.11; *r*_year 2019_ = 0.01; *r*_year 2021_ = 0.28).

The estimated percentage of recorded fruit ripening of *P. majus* and the percentage of faecal samples containing their seed were highly positively correlated with sampling date (*r*_fruit ripening_ = 0.99; *r*_seed occurrence_ = 0.72; Figure 3). In addition, the estimated percentage of *P. majus* fruit ripening and percentage of seed occurrence in faeces over the sampling season were positively correlated (*r* = 0.61).

## 4 Discussion

Fruits are an important food resource for many migratory birds during autumn, but their availability and their presence in the diet of migrants in temperate zones during spring migration have so far been overlooked. Here, we provide quantitative data on fruit availability and consumption of *Prasium majus* and *Rhamnus alaternus* during avian spring migration on Ponza island, a main Mediterranean stopover site. We recorded seed occurrence of *P. majus* and/or *R. alaternus* in faeces of six passerine species and show that within the sampled species, garden warblers had the highest percentage of faecal seed occurrence (Table 1). Furthermore, we found a relationship between seed presence in faeces and individual body condition. We show that *P. majus* seed occurrence in faecal matter, as well as the estimated ripening stage of *P. majus* fruits, were positively related to the progressing date of the season (Figure 3).

*P. majus* and *R. alaternus* are endemic, widespread plants of Mediterranean coastal ecosystems, but their fruit production in spring has not gained much attention. Both plants are dependent on endozoochory for seed dispersal, and their fruits have been proved attractive to birds (Costa et al. 2014; La Mantia et al. 2019). We found that especially *P. majus* has an early fruiting period in April to May, which coincides well with the peak passage time of a range of long‐distance migrants *en route* to their breeding sites in Europe (Maggini, Cardinale, et al. 2020). In contrast, *R. alaternus* fruits reach their peak ripening state mostly in the months of June–July (Bas et al. 2005), 1–2 months after most birds have passed Mediterranean stopover sites on their northward journey (Maggini, Cardinale, et al. 2020). In fact, we only rarely detected fully ripe *R. alaternus* fruits during spring migration passage. We show that *P. majus* and *R. alaternus* seeds were commonly found in passerine faecal samples, and in the case of the garden warbler, seeds were present in up to ~77% of samples collected in one spring migration season (2019). Generally, our data show that *P. majus* fruits are ingested more frequently than *R. alaternus* fruits, probably because of their riper state during birds' stopover (Table 1). Additionally, our data showing differences in *P. majus* faecal seed occurrence across sampling years may hint at ripe fruit availability varying across years, possibly caused by climatic differences between springs (see Section 3 of the Appendix S1, Figures A15 and A16, for weather data between seasons). Long‐term monitoring of the fruit ripening of *P. majus* is required to draw more light on how the plants' fruiting is influenced by weather and how well peak ripening times coincide with passerine spring arrival on Ponza.

We found a lower *P. majus* seed occurrence in faeces of garden warblers with higher body condition (Figure 4). The same trend was evident when considering all tested species which had ingested *P. majus*. Contrastingly, faecal matter from garden warblers with higher body condition was more likely to contain *R. alaternus* seeds (Figure 4). These findings may point to fruits having different nutritional values with dissimilar benefits according to birds' body condition. Furthermore, the larger availability of ripe *P. majus* fruits compared to ripe *R. alaternus* fruits during the migration season also means that birds need to invest less time in finding ripe *P. majus* fruits, making *P. majus* an easily accessible food resource for individuals in a poorer body condition. In addition, we observed that *P. majus* had a thin, easily breakable skin, facilitating a rapid ingestion of the fruit contents. Past research has shown that migratory timing is often correlated with individual body condition, meaning that it is not uncommon to observe seasonal gradients in arrival and departures to/from stopover sites according to birds' fuel stores (Schmaljohann and Naef‐Daenzer 2011; Smolinsky et al. 2013; Smith and McWilliams 2014). An alternative explanation for the correlation between individual body condition and fruit consumption may therefore be that birds in a worse body condition simply arrived later on Ponza and therefore had better access to a larger number of ripe fruits. Yet, we did not find any statistically meaningful relationships between passerine body condition and capture date in our data.

Fruits of *P. majus* and *R. alaternus* contain a variety of nutritional compounds that may support migratory songbirds during energetically demanding flights. *R. alaternus* is rich in anthocyanins (Longo et al. 2005), while phenolic extracts of *P. majus* contain high levels of antioxidants including lutein, ß‐carotene, tocopherols, and polyphenols (Vardavas et al. 2006; Chaouche et al. 2013). Migratory flight is known to induce oxidative stress (Jenni‐Eiermann et al. 2014; McWilliams et al. 2021), and dietary antioxidants may help birds mitigate these effects either prophylactically or postflight (Skrip and McWilliams 2016). Experimental studies have shown that passerines can selectively forage on antioxidant‐rich foods (Bolser et al. 2013; Schaefer et al. 2008), suggesting that antioxidant content may influence fruit selection at stopover sites. Furthermore, *R. alaternus* fruits contain high carbohydrate levels (Herrera 1987) and substantial pulp water content (Bas et al. 2006), which may aid in energy replenishment and rehydration. Although the sugar content of *P. majus* fruits is not quantified, personal field observations suggest a sweet taste, indicating the presence of sugars. Another important nutritional aspect of *P. majus* and *R. alaternus* is polyunsaturated fatty acids such as linoleic and alpha‐linolenic acid (Slezacek et al., unpublished data), which have been associated with improved endurance and metabolic efficiency in birds (Guglielmo 2018; Carter et al. 2020). These nutritional traits may be particularly relevant during migratory stopovers in resource‐limited environments such as Ponza island. We also observed birds feeding on unripe *R. alaternus* fruits; while unripe fruits may contain higher levels of phenols and nitrogen (Schaefer et al. 2003), a more straightforward explanation could be the limited food availability on Ponza (Maggini, Trez, et al. 2020), which may compel birds to consume suboptimal resources, as documented in other frugivorous species under similar conditions (Leck 1972; Foster 1977).

The here reported spatiotemporal bird‐plant interaction in spring (especially between garden warblers and *P. majus* ) most likely represents mutual interest: For migratory birds passing in the fruiting period of *P. majus*, the plants' fruits present an easily accessible food resource, that is, no hunting is required to feed on the fruits, plants carry large numbers of fruits, and they provide a nutritionally valuable refuelling opportunity on a temperate zone stopover site. For *P. majus* on the other hand, the seed rain potential is high when considering the quantity of migratory birds passing through Ponza every spring (Maggini, Trez, et al. 2020; Maggini, Cardinale, et al. 2020). We also noticed that all the seeds collected from bird faeces were unharmed, meaning that none of the bird species that were tested for faecal seed presence in this study seem to be seed predators and seed germination is often enhanced by avian gut passage (Jordaan et al. 2011). More importantly, in times of climate change, plants are urged to disperse northward to track their most suitable ecological niches, providing the most viable environment (González‐Varo et al. 2021). A potential northward dispersal of seeds might represent a further benefit for *P. majus* , making a synchronisation of fruit ripening with peak spring passage of birds advantageous.

## 5 Conclusion

To the best of our knowledge, we here provide the first evidence on the importance of ripe and unripe fruits of *Prasium majus* and *Rhamnus alaternus* as a refuelling opportunity for passerines during spring migration at a temperate zone stopover site. It is still unclear which nutritional factors make particularly *P. majus* and *R. alaternus* fruits attractive to migrants, and further studies are needed to understand their exact nutritional benefit. As long‐term ringing data have shown that birds do not stay on Ponza island for extended periods (Maggini, Trez, et al. 2020), and spring migration is generally characterised by time‐minimising strategies (Kokko 1999), frugivory during this season may serve a more immediate refuelling purpose compared to autumn, when birds often spend longer periods feeding on fruit. Spring migration frugivory on Ponza island could reflect the same underlying need for energy acquisition, adapted to a more time‐constrained migratory context. In this sense, easily acquirable fruits such as *P. majus* may offer a low‐cost, rapid energy source that supports continued migration toward the mainland, where birds may find higher quality food resources. Fruits may further provide easy means for birds to acquire dietary antioxidants, helping to increase antioxidant capacity and maintain oxidative balance during migration (Skrip and McWilliams 2016). For plants, the synchronisation of fruiting with avian spring migration represents a way to direct their seed dispersal northward, a key strategy to cope with climatic consequences of global warming (González‐Varo et al. 2021).

## Author Contributions

**Julia Slezacek:** conceptualization (lead), data curation (lead), formal analysis (lead), investigation (equal), methodology (equal), project administration (equal), visualization (lead), writing – original draft (lead). **Benjamin Kostner:** conceptualization (lead), data curation (equal), formal analysis (equal), investigation (lead), methodology (equal), writing – original draft (lead). **Chiara Agabiti:** data curation (equal), formal analysis (equal), investigation (equal), visualization (equal), writing – original draft (equal). **Massimiliano Cardinale:** investigation (equal), resources (equal), writing – original draft (equal). **Leonida Fusani:** conceptualization (lead), funding acquisition (lead), investigation (equal), methodology (equal), project administration (lead), resources (lead), supervision (lead), writing – original draft (equal).

## Conflicts of Interest

The authors declare no conflicts of interest.

## Supporting information


**Data S1:** ece372239‐sup‐0001‐Supinfo1.txt.

**Data S2:** ece372239‐sup‐0002‐Supinfo2.csv.

**Data S3:** ece372239‐sup‐0003‐Supinfo3.csv.

**Data S4:** ece372239‐sup‐0004‐Supinfo3.txt.

**Appendix S1:** ece372239‐sup‐0005‐Appendix.docx.

## Data Availability

All the required data are uploaded as Supporting Information.
