# Supplementary material for: Berries to Go: Distinct Passerine Spring Migration Frugivory at a Main Mediterranean Stopover Site
Source: Ecol Evol. 2025 Oct 2;15(10):e72239. doi: 10.1002/ece3.72239 (PMC12490958; doi:10.1002/ece3.72239)
Supplement: Supplementary file 5 — Appendix S1: ece372239‐sup‐0005‐Appendix.docx. [file ECE3-15-e72239-s005.docx]

**Appendix**

**1. PCA outputs by species**

*Eurasian blackcap*

PCA standard deviation and variance:

PC1 PC2 PC3

Standard deviation 1.4222 0.8464 0.51098

Proportion of Variance 0.6742 0.2388 0.08703

Cumulative Proportion 0.6742 0.9130 1.00000

PCA eigenvalues:

eigenvalue variance.percent cumulative.variance.percent

Dim.1 2.0225348 67.417826 67.41783

Dim.2 0.7163666 23.878888 91.29671

Dim.3 0.2610986 8.703287 100.00000

PCA loadings:

PC1 PC2 PC3

muscle 0.4660180 0.8847370 0.008228098

fat 0.6264653 -0.3233828 -0.709200087

cond 0.6247947 -0.3356546 0.704959244


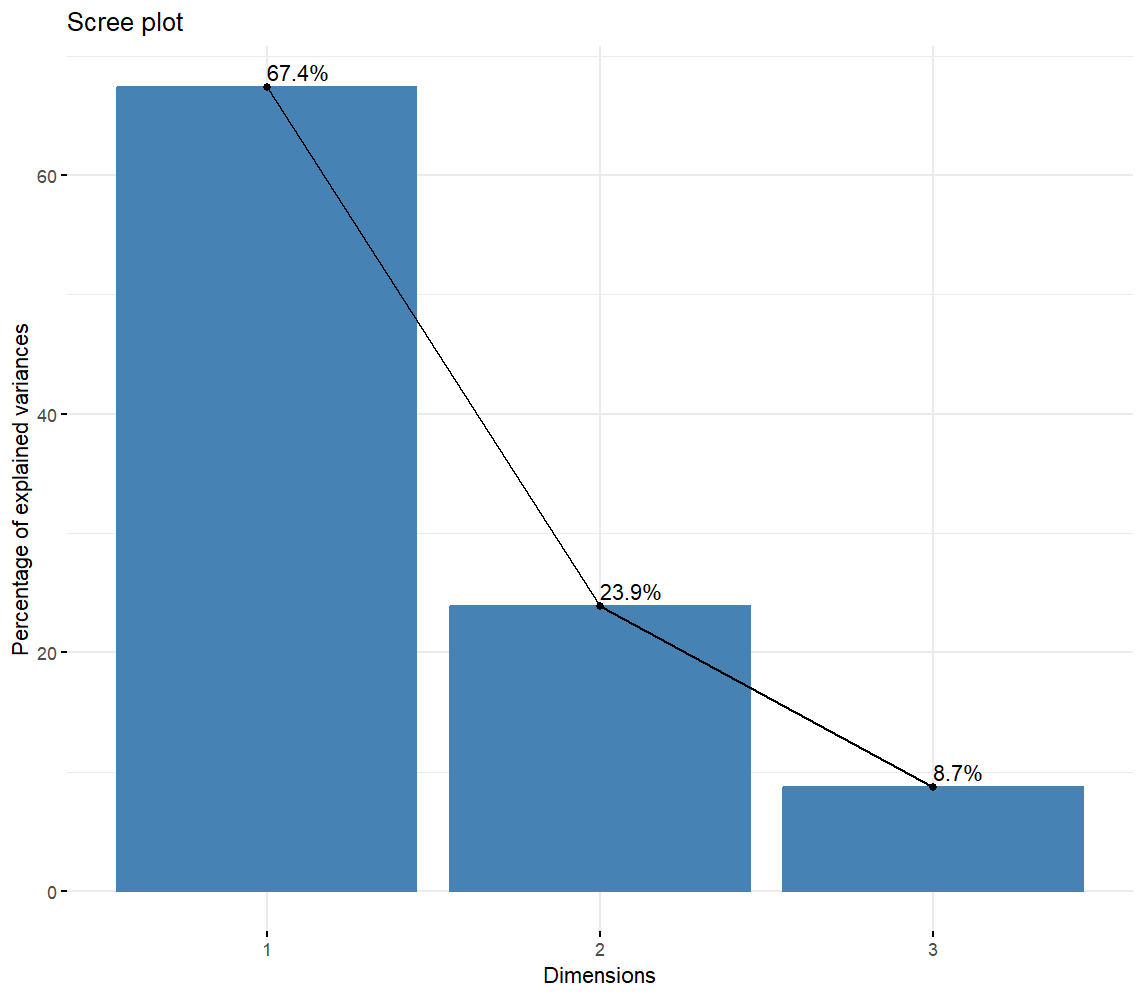


Figure A1. A scree plot depicting percentage of explained variance in PC1 – PC3 in Eurasian blackcaps (Sylvia atricapilla).

*Common redstart*

PCA standard deviation and variance:

PC1 PC2 PC3

Standard deviation 1.4210 0.7778 0.6131

Proportion of Variance 0.6731 0.2017 0.1253

Cumulative Proportion 0.6731 0.8747 1.0000

PCA eigenvalues:

eigenvalue variance.percent cumulative.variance.percent

Dim.1 2.0191576 67.30525 67.30525

Dim.2 0.6049943 20.16648 87.47173

Dim.3 0.3758481 12.52827 100.00000

PCA loadings:

PC1 PC2 PC3

muscle 0.5286199 -0.8484299 -0.02697472

fat 0.6020814 0.3523513 0.71648205

cond 0.5983802 0.3949877 -0.69708381


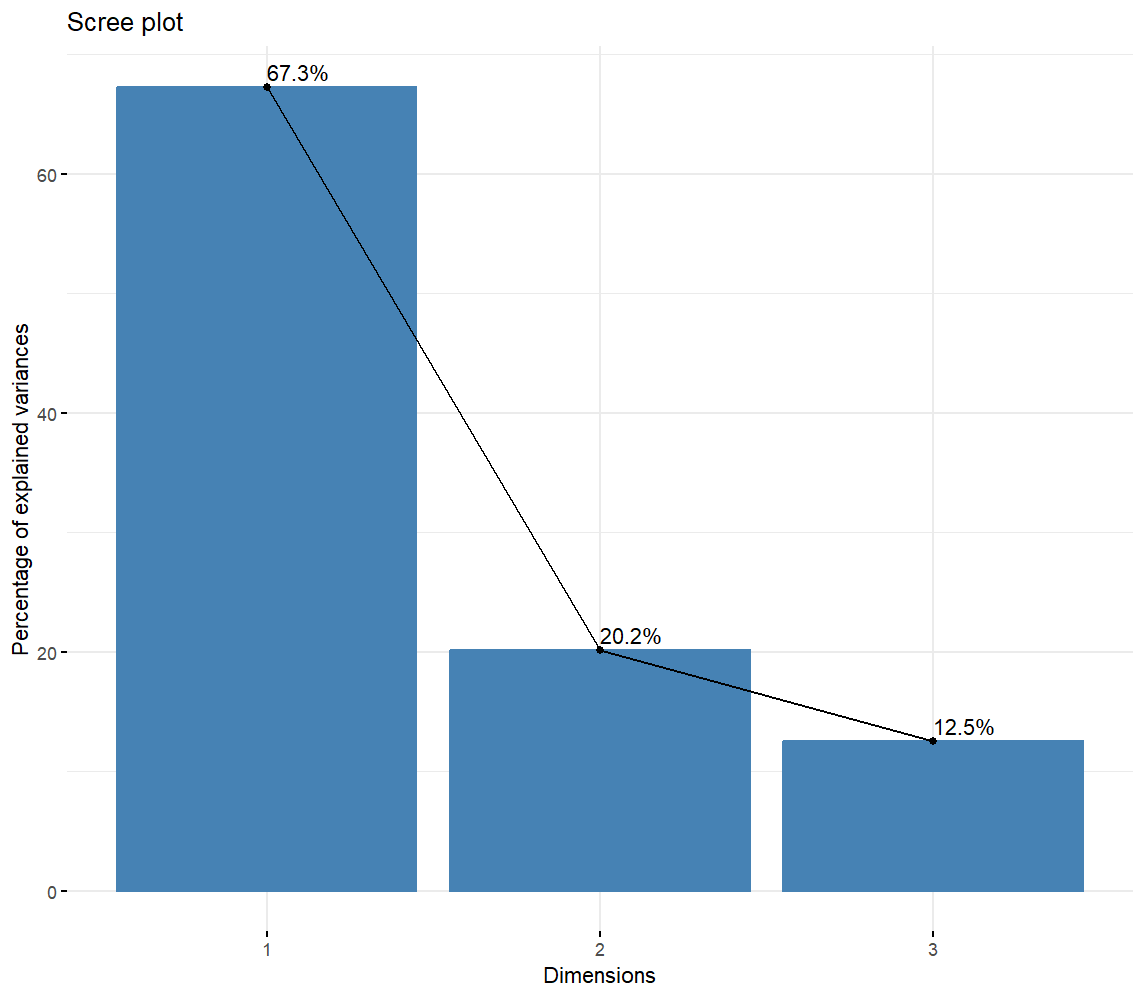


Figure A2. A scree plot depicting percentage of explained variance in PC1 – PC3 in common redstarts (Phoenicurus phoenicurus).

*Garden warbler*

PCA standard deviation and variance:

PC1 PC2 PC3

Standard deviation 1.4081 0.8323 0.5697

Proportion of Variance 0.6609 0.2309 0.1082

Cumulative Proportion 0.6609 0.8918 1.0000

PCA eigenvalues:

eigenvalue variance.percent cumulative.variance.percent

Dim.1 1.9827872 66.09291 66.09291

Dim.2 0.6926960 23.08987 89.18277

Dim.3 0.3245168 10.81723 100.00000

PCA loadings:

PC1 PC2 PC3

muscle 0.4892766 0.8697383 -0.06452691

fat 0.6227427 -0.2966114 0.72402564

cond 0.6105734 -0.3944325 -0.68674824


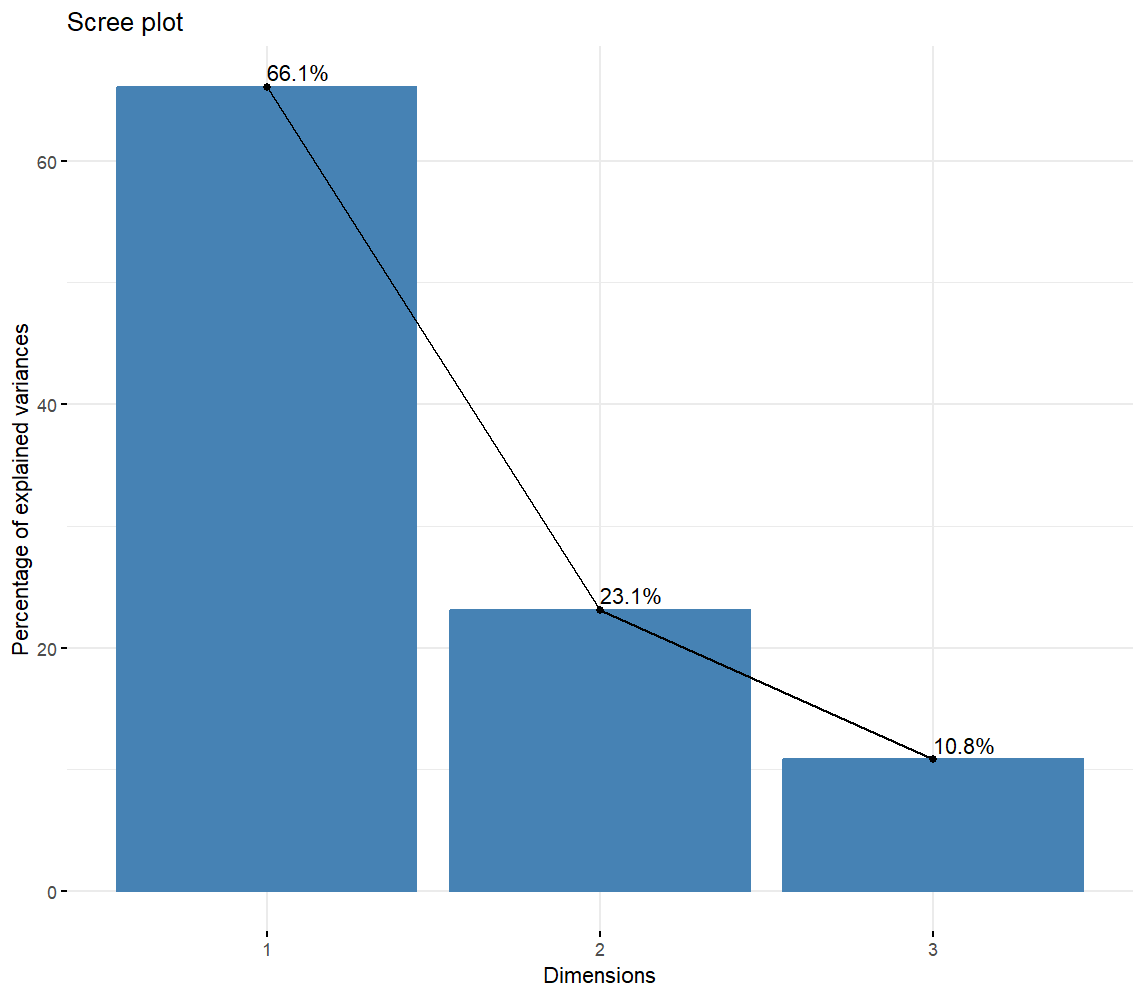


Figure A3. A scree plot depicting percentage of explained variance in PC1 – PC3 in garden warblers (Sylvia borin).

*Icterine warbler*

PCA standard deviation and variance:

PC1 PC2 PC3

Standard deviation 1.3978 0.8105 0.6240

Proportion of Variance 0.6513 0.2190 0.1298

Cumulative Proportion 0.6513 0.8702 1.0000

PCA eigenvalues:

eigenvalue variance.percent cumulative.variance.percent

Dim.1 1.9537976 65.12659 65.12659

Dim.2 0.6568820 21.89607 87.02265

Dim.3 0.3893205 12.97735 100.00000

PCA loadings:

PC1 PC2 PC3

muscle 0.5144207 -0.8573570 -0.01761601

fat 0.6077730 0.3500224 0.71280875

cond 0.6049656 0.3773901 -0.70113719


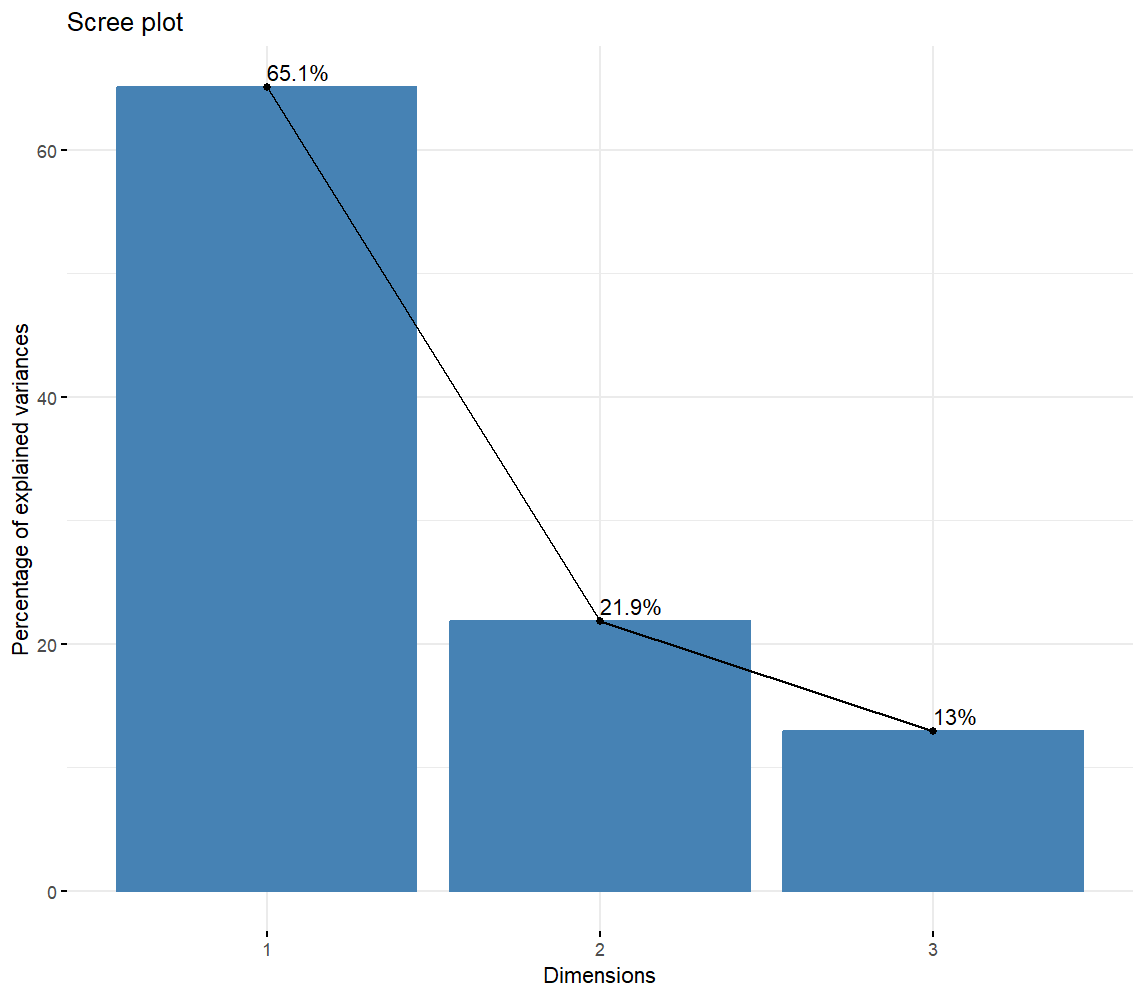


Figure A4. A scree plot depicting percentage of explained variance in PC1 – PC3 in icterine warblers (Hippolais icterina).

*Whinchat*

PCA standard deviation and variance:

PC1 PC2 PC3

Standard deviation 1.3359 0.8111 0.7467

Proportion of Variance 0.5948 0.2193 0.1858

Cumulative Proportion 0.5948 0.8142 1.0000

PCA eigenvalues:

eigenvalue variance.percent cumulative.variance.percent

Dim.1 1.7845632 59.48544 59.48544

Dim.2 0.6578809 21.92936 81.41480

Dim.3 0.5575559 18.58520 100.00000

PCA loadings:

PC1 PC2 PC3

muscle 0.5510560 -0.8343951 -0.01104839

fat 0.5895699 0.3986687 -0.70247461

cond 0.5905461 0.3805890 0.71162304


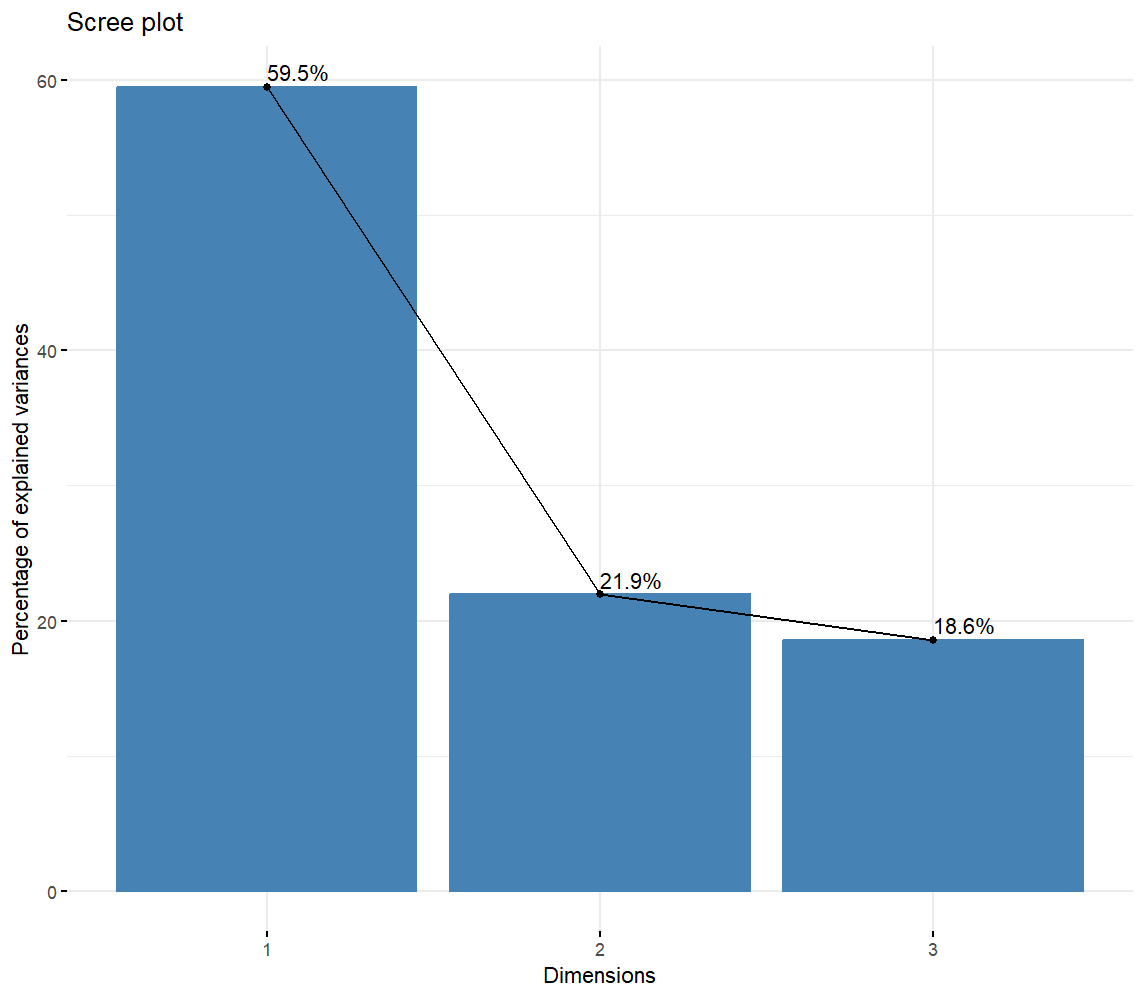


Figure A5. A scree plot depicting percentage of explained variance in PC1 – PC3 in whinchats (Saxicola rubetra).

*Common whitethroat*

PCA standard deviation and variance:

PC1 PC2 PC3

Standard deviation 1.4459 0.8085 0.50563

Proportion of Variance 0.6969 0.2179 0.08522

Cumulative Proportion 0.6969 0.9148 1.00000

PCA eigenvalues:

eigenvalue variance.percent cumulative.variance.percent

Dim.1 2.0906556 69.688520 69.68852

Dim.2 0.6536810 21.789368 91.47789

Dim.3 0.2556634 8.522112 100.00000

PCA loadings:

PC1 PC2 PC3

muscle 0.4909557 -0.8711185 0.01072358

fat 0.6150244 0.3552884 0.70392839

cond 0.6170150 0.3390024 -0.71019000


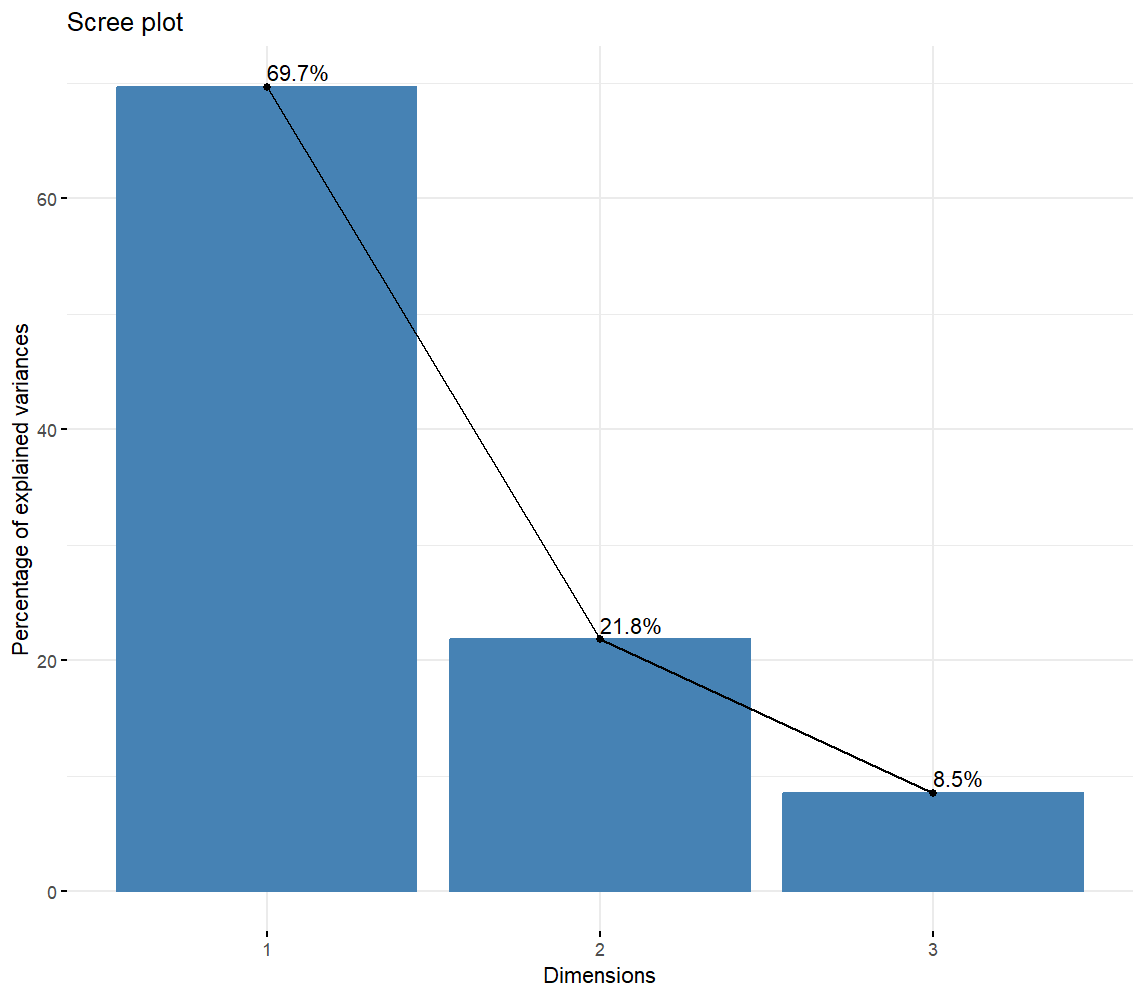


Figure A6. A scree plot depicting percentage of explained variance in PC1 – PC3 in common whitethroats (Curruca communis).

**2. Model diagnostics**

*Faecal seed occurrence of Prasium majus in garden warblers*

GLM: faecal P. majus seed occurrence ~ body condition + year + day of May, family = binomial


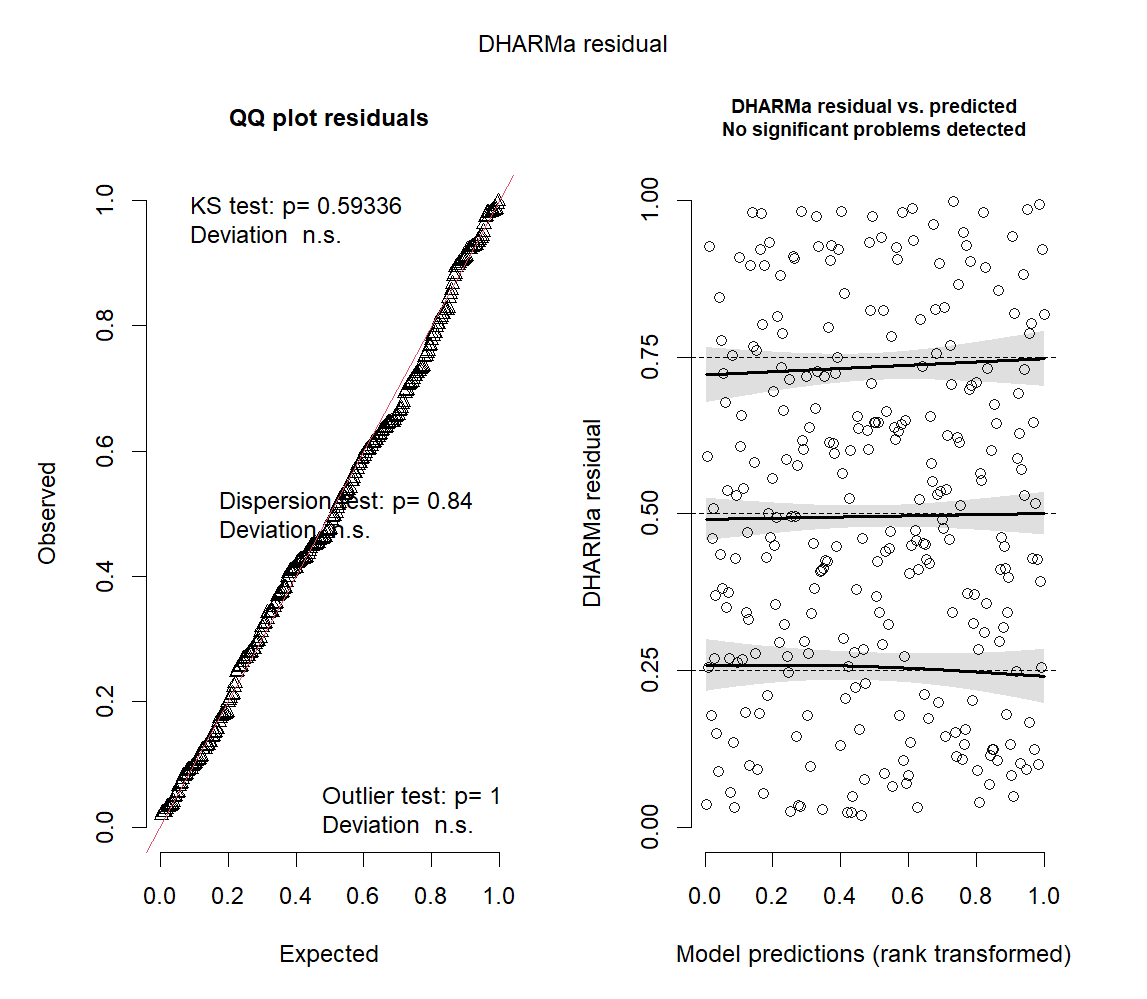


Figure A7. DHARMa residual QQ plot and residuals vs. predicted plots.


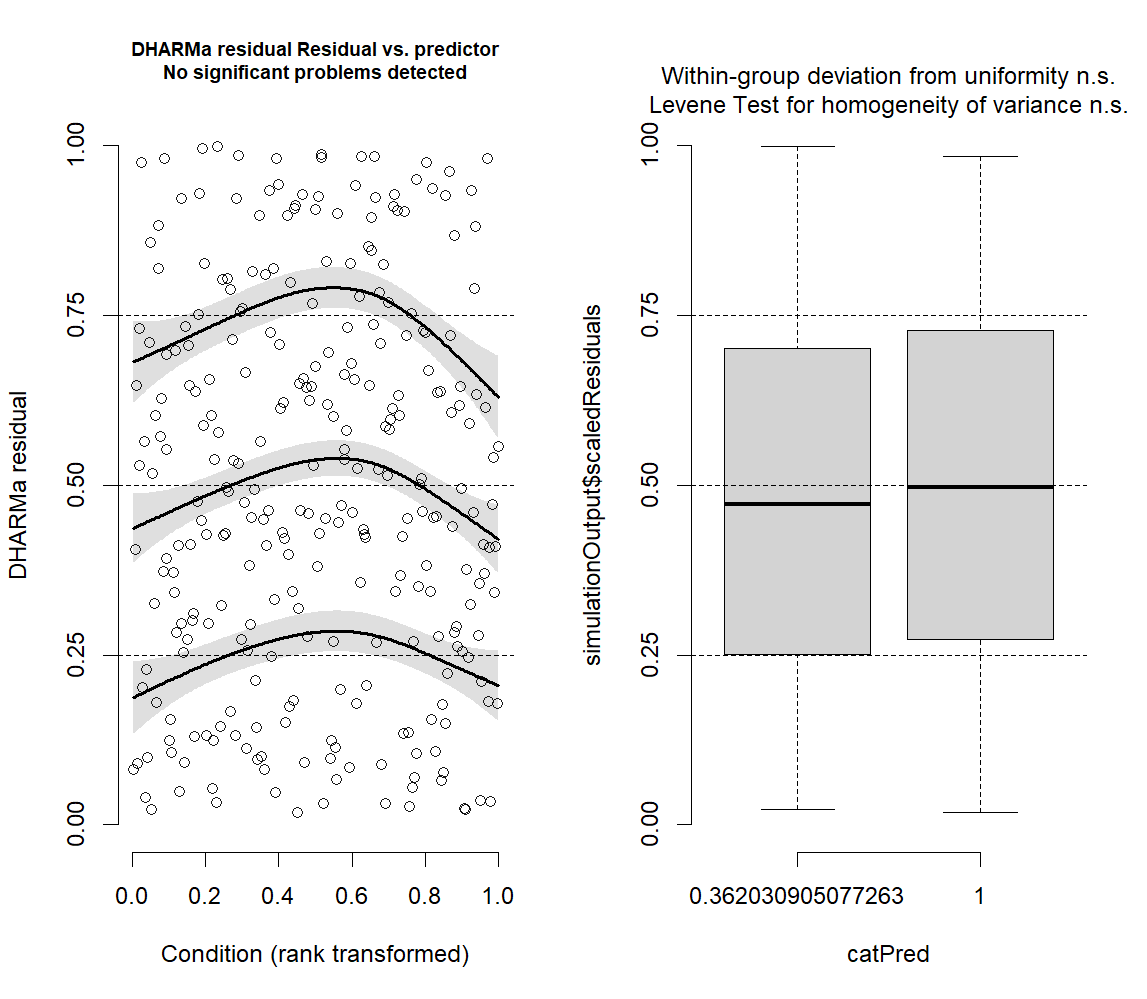


Figure A8. DHARMa residual plots for the independent variables body condition (left) and year (right).


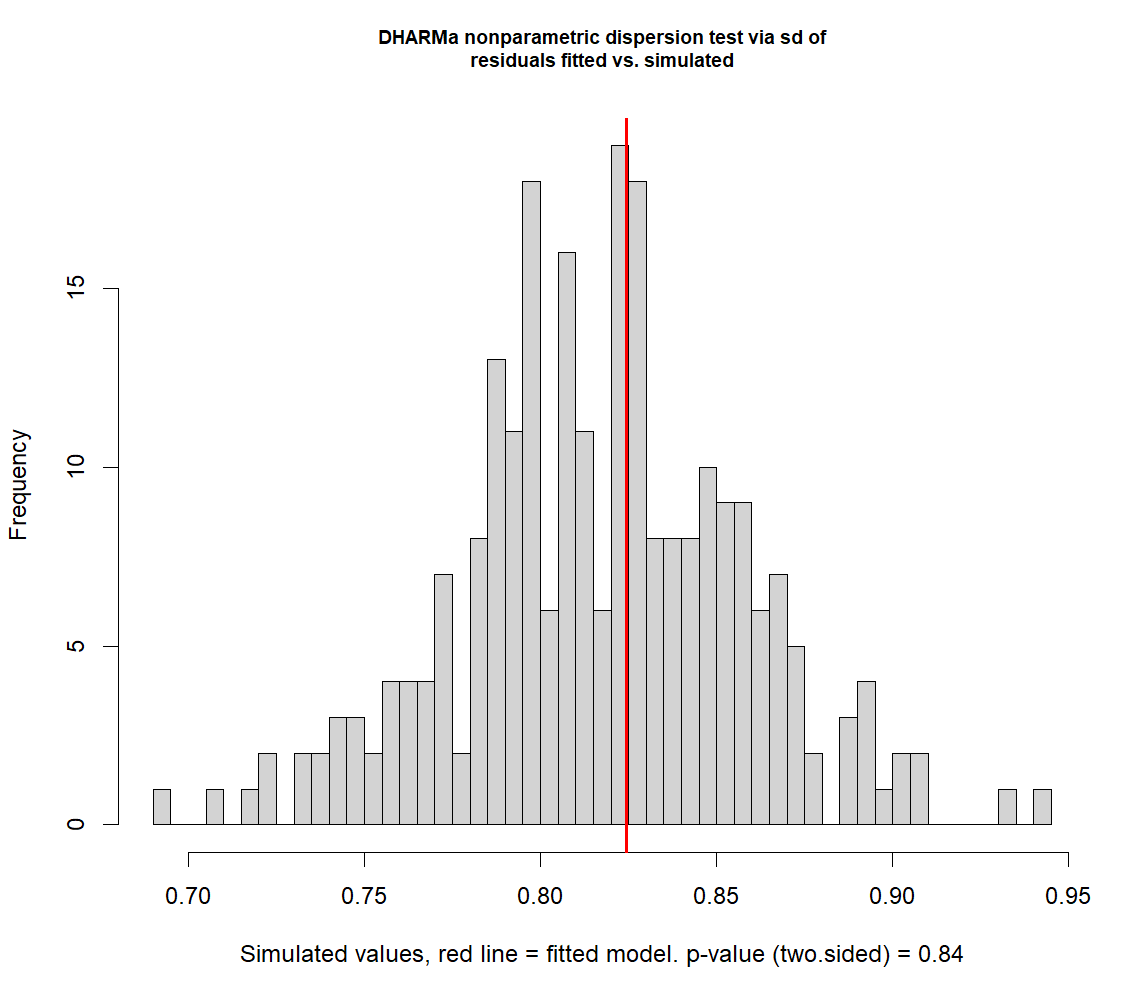


Figure A9. DHARMa dispersion test.

*Faecal seed occurrence of Rhamnus alaternus in garden warblers*

GLM: faecal R. alaternus seed occurrence ~ body condition + year + day of May, family = binomial


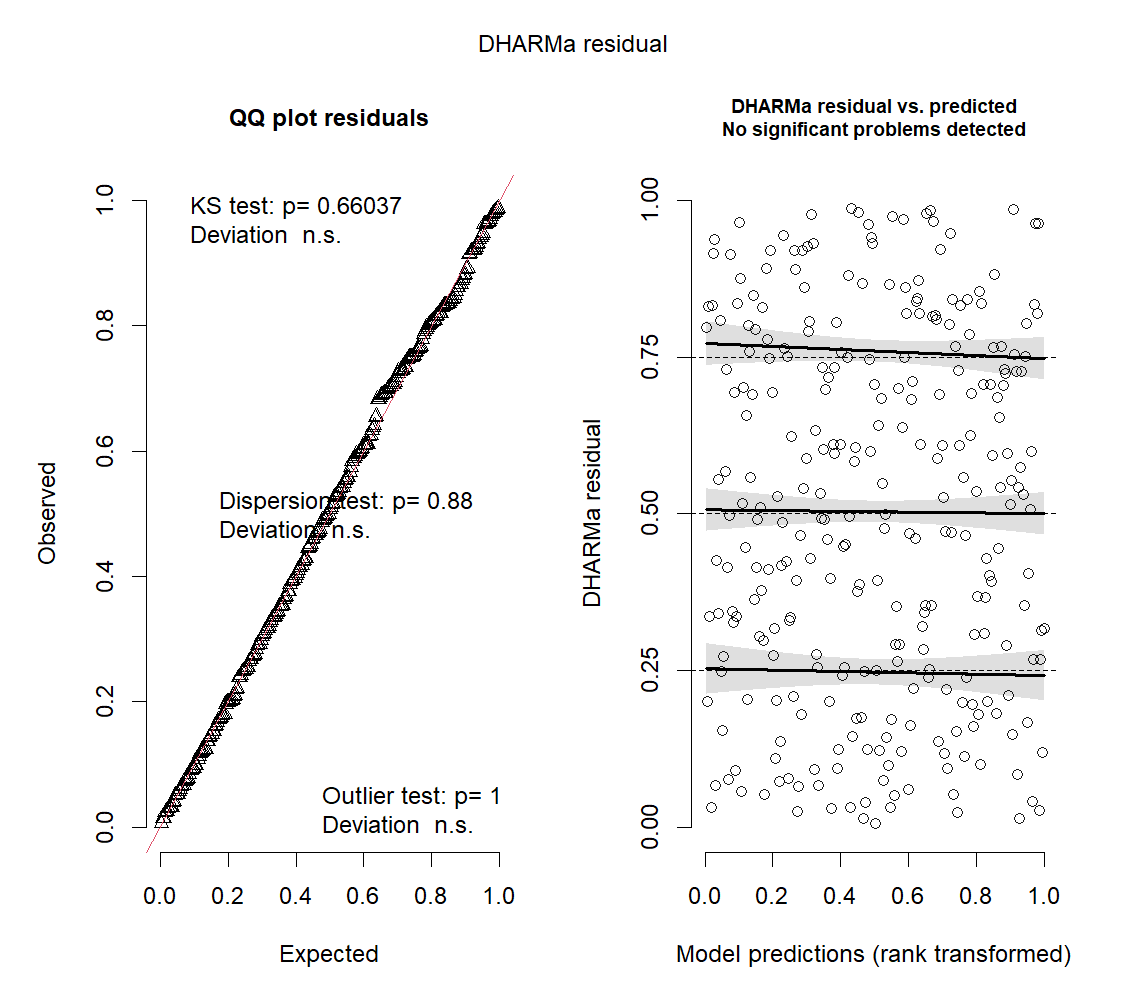


Figure A10. DHARMa residual QQ plot and residuals vs. predicted plots.


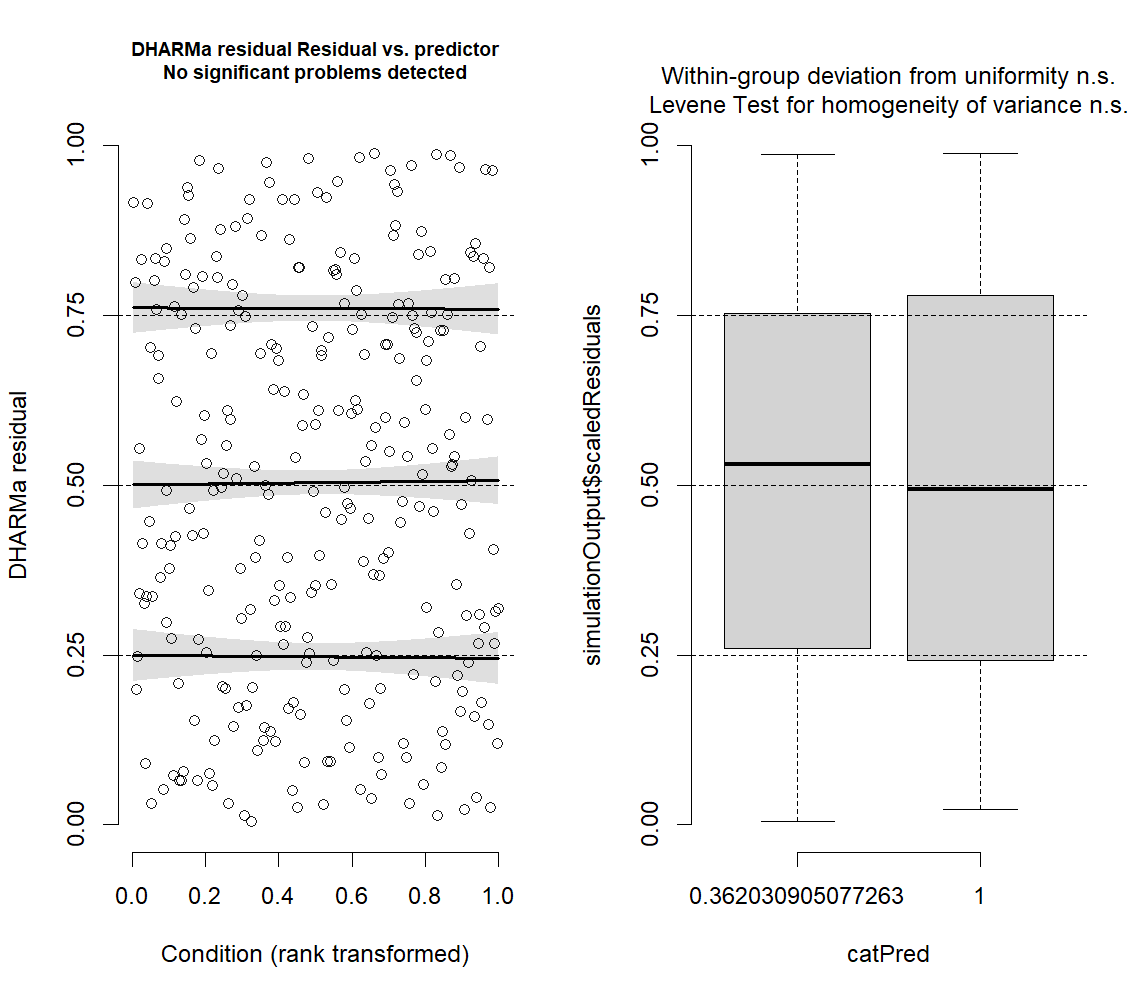


Figure A11. DHARMa residual plots for the independent variables body condition (left) and year (right).


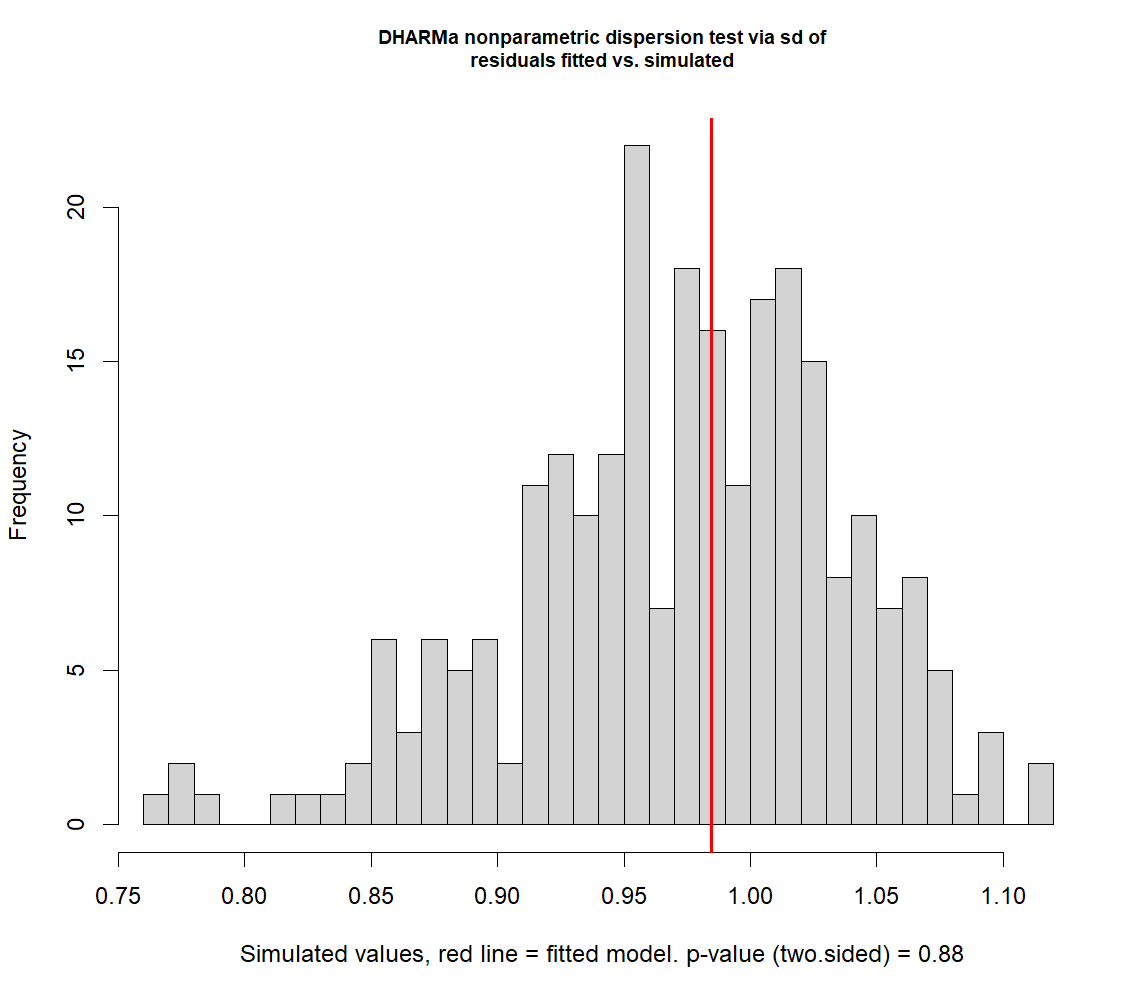


Figure A12. DHARMa dispersion test.

*Comparison of faecal seed occurrence of prasium majus across sampled bird species*

GLM: faecal P. majus seed occurrence ~ body condition + year + species, family = binomial


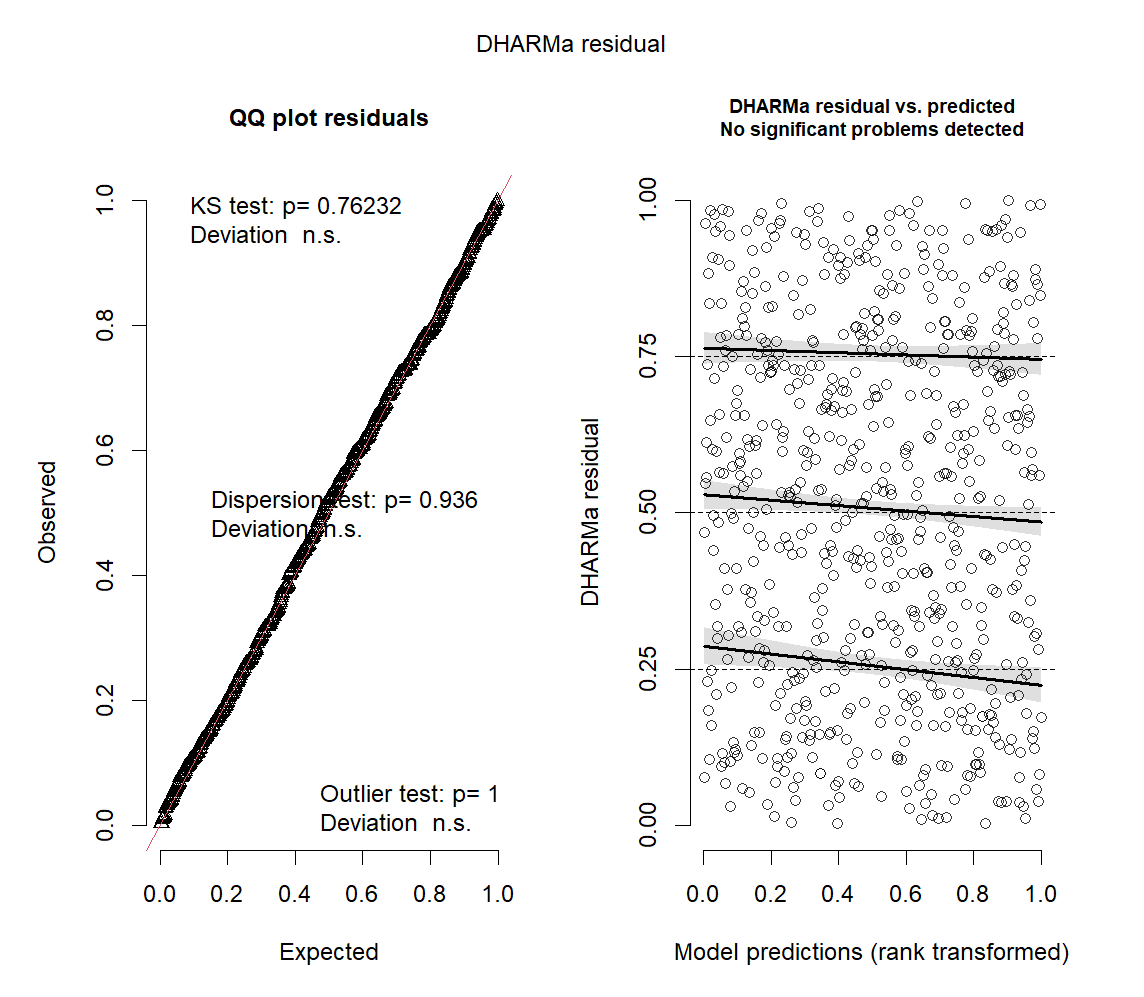


Figure A13. DHARMa residual QQ plot and residuals vs. predicted plots.


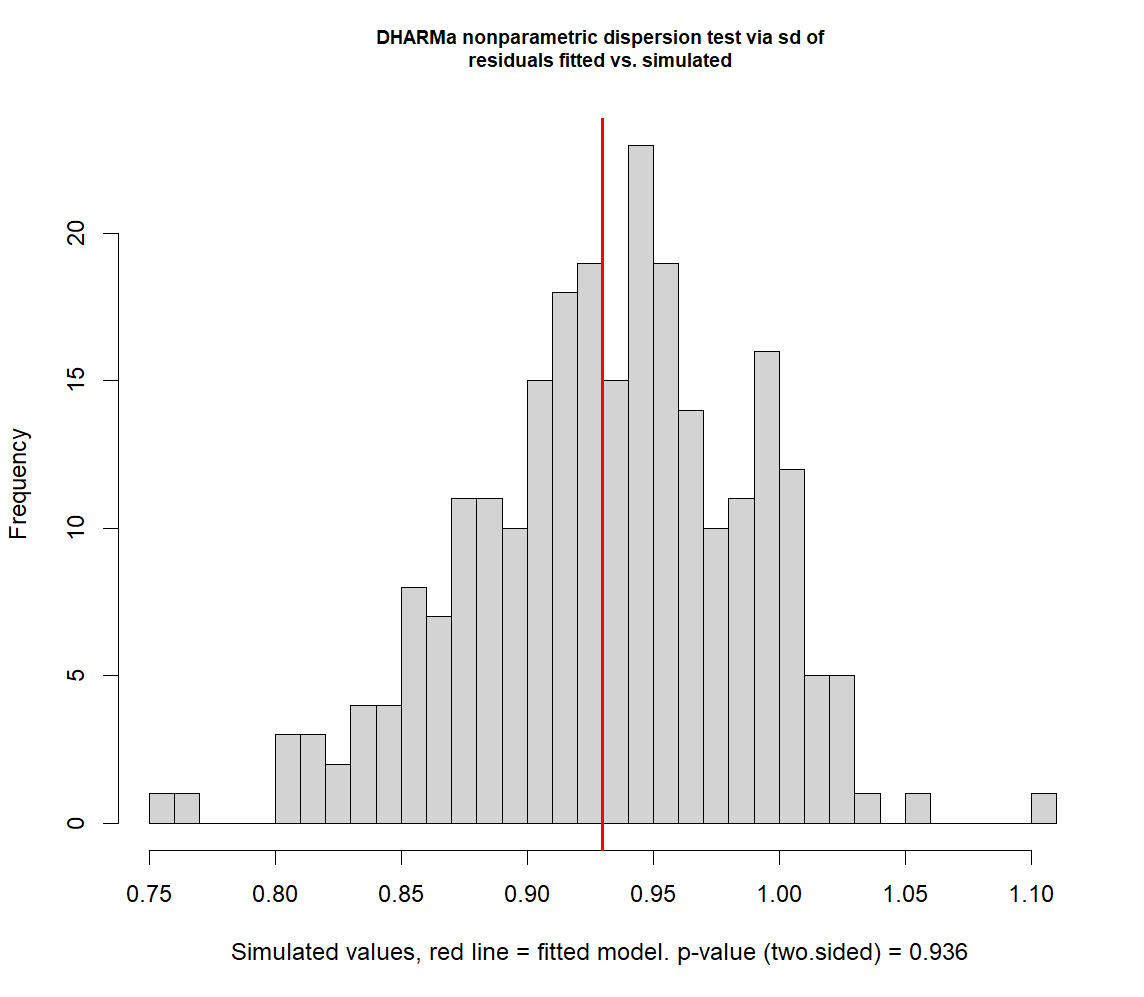


Figure A14. DHARMa dispersion test.

**3. Weather parameters for spring of 2019 and 2021 on the island of Ponza**

We used weather data from https://www.worldweatheronline.com/ponza-weather-history/lazio/it.aspx to create datasets on ambient temperatures and rainfall in both sampling seasons. We used temperature minima, maxima, and averages to compare ambient temperatures between the seasons in Wilcoxon’s rank sum tests as the data were not normally distributed. We analysed data on average daily rainfall in the same way.

Temperature minima (Wilcoxon’s W = 4729, *p* < 0.01; figure A15.), maxima (Wilcoxon’s W = 4022, *p* = 0.01; figure A15.), and averages (Wilcoxon’s W = 4483.5, *p* < 0.01; figure A15.) were significantly lower in the field season of 2021 compared to 2019 but there was no significant difference in average rainfall between the seasons (Wilcoxon’s W = 3242.5, *p* = 0.89; figure A16.).


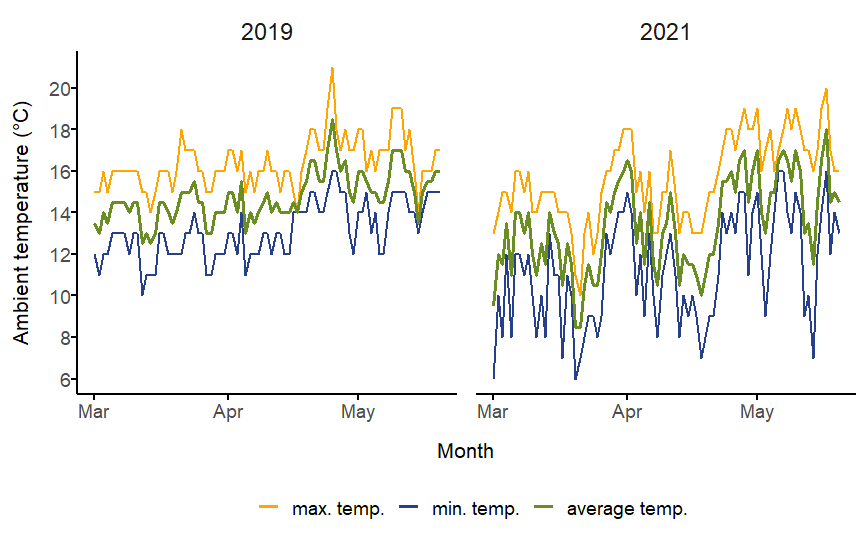


*Figure A15. Ambient temperature averages, minima, and maxima on the island of Ponza from March 01^st^ to May 20^th^ in the years 2019 and 2021.*


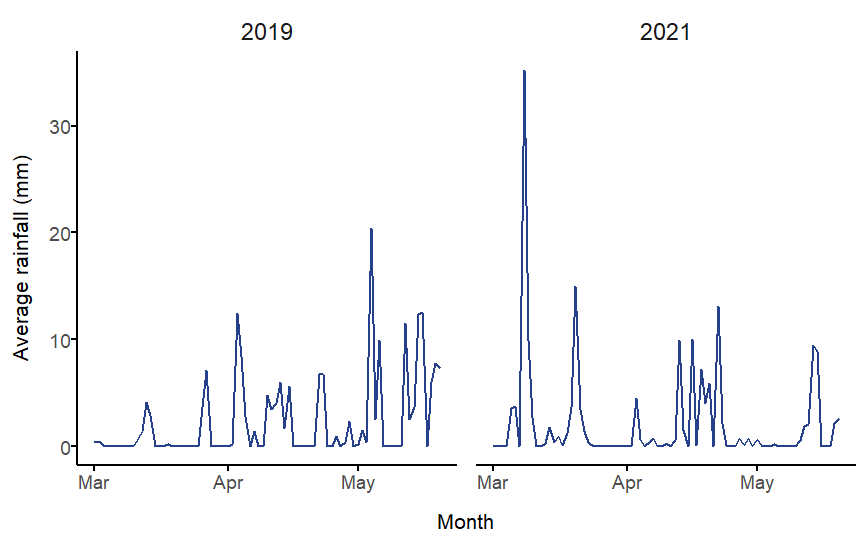


*Figure A16. Average rainfall between the years 2019 and 2020 (March 01^st^ to May 20^th^) on Ponza Island.*
